# Problems identified by dual sensory impaired older adults in long-term care when using a self-management program: A qualitative study

**DOI:** 10.1371/journal.pone.0173601

**Published:** 2017-03-21

**Authors:** Lieve Roets-Merken, Sytse Zuidema, Myrra Vernooij-Dassen, Marianne Dees, Pieter Hermsen, Gertrudis Kempen, Maud Graff

**Affiliations:** 1 Donders Institute for Cognition, Brain and Behaviour, Radboud Institute for Health Sciences, Radboud University Medical Center, Nijmegen, The Netherlands; 2 Kalorama Foundation, Beek-Ubbergen, The Netherlands; 3 Department of General Practice and Elderly Care Medicine, University Medical Center Groningen, University of Groningen, Groningen, The Netherlands; 4 IQ Healthcare, Radboud Institute for Health Sciences, Radboud University Medical Center, Nijmegen, The Netherlands; 5 Severinus Foundation, Veldhoven, The Netherlands; 6 Department of Health Services Research, CAPHRI School for Public Health and Primary Care Maastricht University, Maastricht, The Netherlands; TNO, NETHERLANDS

## Abstract

**Objective:**

To gain insights into the problems of dual sensory impaired older adults in long-term care. Insights into these problems are essential for developing adequate policies which address the needs of the increasing population of dual sensory impaired older adults in long-term care.

**Methods:**

A qualitative study was conducted in parallel with a cluster randomized controlled trial. Dual sensory impaired older adults in the intervention group (n = 47, age range 82–98) were invited by a familiar nurse to identify the problems they wanted to address. Data were taken from the semi-structured intervention diaries in which nurses noted the older adults’ verbal responses during a five-month intervention period in 17 long-term care homes across the Netherlands. The data were analyzed using descriptive statistics and qualitative content analysis based on the Grounded Theory.

**Findings:**

The 47 dual sensory impaired older adults identified a total of 122 problems. Qualitative content analysis showed that the older adults encountered participation problems and problems controlling what happens in their personal environment. Three categories of participation problems emerged: (1) existential concerns of not belonging or not being able to connect with other people, (2) lack of access to communication, information and mobility, and (3) the desire to be actively involved in care delivery. Two categories of control-in-personal-space problems emerged: (1) lack of control of their own physical belongings, and (2) lack of control regarding the behavior of nurses providing daily care in their personal environment.

**Conclusions:**

The invasive problems identified indicate that dual sensory impaired older adults experience great existential pressures on their lives. Long-term care providers need to develop and implement policies that identify and address these problems, and be aware of adverse consequences of usual care, in order to improve dual sensory impaired residents’ autonomy and quality of life.

## Introduction

The prevalence of dual sensory impairment (DSI) among older adults in long-term care (LTC) facilities has increased rapidly from 12% in 2005 to 33.9% in 2016 and is expected to continue to grow strongly [1, 2]. DSI has been linked to an augmented risk of depressive feelings, cognitive decline and mortality, and to decreased functional independence, and limited communication and interaction [3–7]. Research showed that DSI residents in LTC who were involved in activities did not have a higher mortality than a comparable group of non-DSI residents, while residents with DSI who were not involved in activities had a 51% higher mortality [8].

Since the new millennium, the LTC-population in the Netherlands has greatly altered; from being a relatively independent 70+ population, it has become an 85+ population suffering from a complex of aged-related diseases, highly dependent on professional care [9]. Consequently, it is not clear if the current LTC-policies towards sensory impairment are still adequate for the current population. For the pre-millennium LTC population, a sensory impairment was primarily an individual problem that could be addressed at an individual level by medical treatment, device provision, and possibly a psychosocial rehabilitation program. After having completed this ‘cure and care’ program, LTC residents with their (often single) sensory impairment, were expected to be able to cope independently and to self-manage daily activities adequately. Therefore, sensory impairment was given low priority in LTC. Until now, expertise centers for aged care and hearing or visual rehabilitation recommend an annual screening of both hearing and vision as best practice, with, if necessary, a referral to relevant medical specialists or device providers, supplemented with nurse attention for correct use of the devices [10, 11].

However, findings from research and practice question the effectiveness and substantiation of the current LTC policies towards the needs of the increasing number of DSI residents with aggravated mental, sensory, and physical conditions. First, a lack of adequate hearing and visual screening has been reported. LTC facilities often overlook and poorly document the incidence of a hearing and/or visual impairment [12, 13]. Furthermore, more often than in the pre-millennium LTC population, the current frail elderly population suffer from an advanced hearing or visual disease such as aged-related macular degeneration, diabetic retinopathy or presbycusis. These diseases cannot always be sufficiently addressed by medical treatment and/or compensated by device provision. In addition, the psychosocial rehabilitation programs that are offered either in combination with or subsequent to hearing aid or low vision device provision, are found to have limited effect for older adults [14]. Finally, whereas a single sensory impairment can be more or less compensated by using the other sense, individuals with a DSI cannot rely on any compensation and therefore need new coping measures and support.

Until now, little is known about the problems and needs which the DSI older adults encounter in LTC in their daily lives. Consequently, little is known about the support and solutions LTC facilities could create to alleviate these problems and improve their quality of life. The aim of this qualitative study is to gain insights into the problems identified by DSI older adults in LTC.

## Methods

### Study design and ethical approval

This qualitative study was conducted in parallel with a cRCT that assessed the effectiveness of a self-management program for dual sensory impaired older adults (the SMP-DSI program) on social participation, and was performed between August 2011 and April 2014. Details of the cRCT methods are available in the study protocol [15]. The study design and protocol of the RCT and connected qualitative studies was approved by the Dutch Committee on Research involving Human Subjects region Arnhem-Nijmegen, ABR 26192.091.08. This qualitative study focused on older adults problems and needs by analyzing the quotes of the older adults’ verbal responses as noted by the nurses in their’ semi-structured intervention diaries, and collected during a five-month SMP-DSI intervention period in 17 long-term care homes across the Netherlands. The data were analyzed using descriptive statistics and qualitative content analysis based on the Grounded Theory. Trial registration: Clinical Trials NCT01217502.

### Study participants

In total, 47 DSI older adults participated in the qualitative study. They were residents of one of the 17 LTC homes allocated to the cRCT intervention group. The inclusion criteria for the residents were (1) aged 55 or over, (2) a hearing impairment of PTA ≥40 dB (best ear) and a visual impairment with a best-corrected visual acuity of ≤0.3 diopter or with a visual field of <30°, measured using the criterion standards for hearing and visual impairment [16, 17] and (3) written informed consent. Exclusion criteria were (1) prelingual deafness, (2) a DSI acquired before the age of 50, and (3) inability to complete interviews due to cognitive problems, as assessed using the DSM IV criteria for capacities in executive functioning [14, 18]. Each eligible older adult was linked to a licensed practical nurse. Prior to the start of the intervention, the DSI older adults were interviewed following the guidelines of the study protocol and interview guide. We collected demographics and characteristics on native language, preferred communication modality, and care dependency when performing daily activities. Care dependency was measured using the Katz-ADL index (bathing, dressing, toileting, transferring and feeding), with scores ranging from 0 to 5 with a higher score indicating more dependency [19].

After inclusion of the eligible DSI older adults, the manager invited nurses familiar with the dual sensory impaired older adult to participate in the study. The nurses (n = 34) who verbally accepted the invitation were linked to one or two DSI older adults. Inclusion criteria for nurses were (1) at least twice-weekly direct daily care contact with the participating older adult, and (2) qualified as a licensed practical nurse, that is, a three-year basic nursing vocational training at secondary level.

In accordance with the consent procedure of the Dutch Committee on Research involving Human Subjects region Arnhem-Nijmegen, the written consent given by the older adults for participation in the hearing and visual measurement, and if eligible, for participation in the trial, were documented in the research database and archived in the research file. The names and contact information of the nurses who were invited by their manager and who verbally consented to participate were documented in the research database.

### Data collection

In individual sessions, the nurses invited their DSI older adults to identify problems, wishes or things they wanted to alter or address. This invitation was the first step of the Self-Management Program for Dual Sensory Impaired older adults (SMP-DSI). In accordance with the design of the problem-solving therapy and self-management programs, nursed did not ask the older adults to restrict their problems to the LTC environment nor to DSI-related issues [20–22].

Nurses were asked to introduce the SMP-DSI to the DSI older adult at least every three weeks over the five-month intervention period and to literally quote the older adults’ verbal responses in a semi-structured intervention diary during or immediately after the interview with the DSI older adult. S1 Table shows the intervention diary and supportive questions. Every two months, research assistants collected the nurses’ intervention diaries and posted them to the research teamer.

### Data analysis

Prior to the analyses, an administrative assistant transcribed each handwritten intervention diary in a digital MSWord document. Each document was anonymized and given a code referring to the older adult and the nurse. To analyze the data qualitative content analysis was performed using the constant comparison approach based on the Grounded Theory [23]. Two authors, each with a different background, LRM (psychologist) and MD (general practitioner) independently analyzed the data through a process of inductive comparison and reasoning, starting from the collected data and not from preexisting theories. After reading and re-reading the transcriptions, the two authors developed the emergent themes following a series of coding steps. Firstly (open coding), initial coding was generated by coding chunks of the transcripts which were given conceptual labels (= codes) closely related to the participants’ words to isolate the basic units of meaning. Both authors compared and discussed these codes until they reached consensus. Next (axial coding), they identified relations between the initial codes and grouped those codes referring to the same phenomenon into categories. Here again, the two authors discussed these categories until consensus was reached. Finally (selective coding), the categories were organized into themes, and discussed until consensus was reached. The results of this inductive analysis were not sent to the DSI older adults or to the nurses; they were sent to the trainers who were then asked to provide written feedback. ATLAS-ti version 7.0.92 software was used during this process of analysis [24].

## Findings

### Demographics

Forty-seven older adults participated in the intervention. Their ages ranged from 82–98 years, with a mean (SD) age of 90.8 years (SD = 4.4). Thirty-four (72.3%) of them were women, and 43 (91.4%) were widowed or lived without partner. They were moderately to severely dependent on support when performing daily activities; the Katz-ADL index (was 3.7 (SD = 1.1). All were native Dutch speakers and their preferred communication modality was speech, none of the participants used tactile communication. Pure-tone audiometry showed that 34 (72.3%) older adults had a moderate hearing loss ranging between 40–60 decibels (average threshold of the frequencies between 1000, 2000 and 4000 Hertz of the best ear), 9 (19.1%) persons had a severe hearing loss ranging between 61–80 decibels, and 4 (8.5%) showed a profound hearing loss ≥81 decibels. Measurements of visual acuity, visual field and self-reported visual problems gave 14 (29.7%) participants with a moderate visual impairment (visual acuity ≥ 0.5–0.3 diopter with additional reading and/or visual field problems and self-reported visual problems), 12 (25.5%) a low vision (visual acuity 0.3–0.05 diopter) and 21 (44.6%) a profound low vision (visual acuity ≤ 0.05 diopter). A total of 258 intervention diaries were collected, showing 122 different problems identified by the DSI older adults. Table 1 presents the results of the qualitative content analysis based on those 122 problems. Two themes of problems emerged from the constant comparison approach: the participation problems and control-in-personal-space (CIPS) problems. Seventy-eight (64%) of the 122 problems belonged to the participation theme and 44 (36%) to the control-in-personal-space theme. Although nurses did not ask to restrict the problems and wishes to the LTC environment, all 122 problems were related to the daily life and care in LTC; not one referred to the family or private social life of the older adults.

**Table 1:** Problems identified by the older adults using the SMP-DSI.

Themes	Categories	Codes
Participation	Existential concerns	Feelings of not belonging; solitude; not being able to connect with others; shame; fear to behave foolishly; emptiness; aimless days; fear of being forgotten
	Lack of access	Not being able to understand what others are saying; not being able to recognize others when passing them in the corridor; not being able to get information about what happens in the surroundings; not being able to orientate in and around the setting
	Care delivery	Negotiation on the planning and organization of care; adjustment of appointments; dissatisfaction with behavior of care staff; dissatisfaction with changes introduced by the management
Control-in-personal-space	Lack of control on physical belongings	Not being able to control medication intake; no overview of the placement of furniture in own apartment; not being able to retrieve personal belongings from cupboard or wardrobe; not being able to use technical devices properly; not being able to identify drinking cups, dinner sets or cutlery
	Lack of control on the support of the nurses	Uncertainty what nurses do with medications; uncertainty what nurses do with personal belongings of the older adult

### Participation problems

The theme participation reflects three categories: (1) the existential concerns of DSI older adults not to be able to belong to, to connect with other people or to behave appropriately (n = 23 problems), (2) the lack of access to communication, information and moving around freely (n = 29), and (3), the desire to be actively involved in care delivery (n = 26). Examples of participation problems identified by the DSI older adults and noted by the nurses in the intervention diaries are:

#### Category Existential concern

P 38: I can’t go to the day care; I feel stupid, I don’t recognize anyone: they might say things to me that I can’t understand.

P 47: You and your colleagues have forgotten me for two days, I was ill in bed, you probably thought I was asleep, I didn’t hear or see you.

#### Category lack of access

P 3: I can’t read the home’s newsletter.

P 12: I find it really difficult to communicate with my neighbor.

P 6: I can’t find my way to the bench outside.

#### Category care delivery

P 33: The doctor is always too late for the appointment; usually they come at lunchtime. Then it takes a lot of energy to get to my room, but I don’t dare tell her.

P 67: I’m not happy with the care plan drawn up for me.

### Control-in-personal-space problems

The theme Control-in-personal-space reflects the lack of control of DSI older adults regarding (1) their own physical belongings (n = 26), and (2) on nurses’ support when handling these belongings and medications during daily care (n = 18). Examples of CIPS problems identified by the DSI older adults and noted by the nurses in the intervention diaries are:

#### Category lack of control on physical belongings

P 56: I’d like to better position the furniture in my room.

P 19: I can’t feel the difference between the front and back of my sweater.

#### Category lack of control on the support of the nurses

P 37: The nurse said something, her name or something like that, put something in my fridge, and quickly went away, I’m not sure who she was and what she did.

P 41: The nurse said that the medicine is the same, but I don’t really believe her, I can feel that they are a different shape.

## Discussion

In this study, qualitative content analysis based on the Grounded Theory identified participation and control-in-personal-space (CIPS) as main problem themes amongst dual sensory impaired (DSI) older adults in long-term care (LTC). Among the participation problems, existential problems like the fear of not being able to connect with others emerged, as well as problems about access to communication, information and moving around freely, and in care delivery. Among the CIPS problems, lack of control over physical belongings and nurse support when manipulating these belongings emerged as issues. All categories of problems were related to daily life and care in the LTC setting. The findings suggest that the DSI older adults feel threatened in their existence as a social human being; they feel unable to reach out to others, to be aware of what is happening in their environment, or to discuss and negotiate about the care they receive. In addition, they feel hindered in using their physical belongings properly, and in controlling the professionals’ support and handling of their belongings.

Our findings are in line with the limited number of studies that have identified either social isolation, decreased functional independence, or problematic communicative interactions as important risks of DSI at an advanced age [5, 7, 25]. However, the qualitative content analysis performed in this study helped produce a more coherent and overarching picture of the problems of DSI in this group. Our findings suggest that DSI is not associated with a single problem, but that a complex of invasive problems threatens the social, mental and physical health of the DSI older adult.

Although the DSI older adults participating in this study differ greatly from deafblind persons who acquired a DSI at an early stage of life, our findings are most consistent with those found in the literature on deafblindness. The term deafblind is primarily reserved for children and younger adults whose development is greatly affected by the early onset of the DSI. The DSI older participants in our study acquired DSI at an advanced age, having led a ‘normal’ life, and therefore being accustomed to participating in a hearing and sighted community and using spoken language. Despite these differences, our finding of participation problems among the DSI older adults is in line with the literature on these deafblind younger adults, who describe participation problems as the main and overarching risk of deafblindness [26–28]. There is a strong similarity between the category lack of access that we found among the older adults, and the three classic domains that are usually aimed at in deafblind rehabilitation, i.e. (1) problems in communication, (2) acquisition of information, and (3), spatial orientation and moving around freely. However, there are some differences. The DSI older adults in our study added existential concerns like fear of not being able to connect, or the fear of behaving inappropriately, to the list of DSI-related problems. In addition, whereas deafblind youngsters attempt to acquire independency through self-help skills, the older adults in this study did not primarily show a desire to regain these skills, but to be able to control their own activities and those of care providers when handling their belongings or medication. In conclusion, whereas the deafblind literature and education concentrate on the development of communication and self-help skills of the younger adults [29], the findings in this study highlight the need to concentrate on and address the existential pressure, including feelings of fear and shame, and on the lack of control that DSI older adults experience.

At first sight, the participation problems found in this study seem in line with the feelings of social isolation found among the frail older adults in LTC [30]. Problems such as loneliness and aimless days may occur among both DSI and non-sensory impaired older adults, but some participation problems identified in this study show a connection with their DSI, so there is a need for an LTC to be cautious when addressing these participation problems. Whereas hearing and sighted older adults can benefit from an LTC environment that offers stimulating group activities to support participation [31], our study shows that DSI older adults may encounter serious barriers when meeting other people, which increases the risk of heightening their feelings of not belonging.

A key lesson that can be learned from our findings on problems such as lack of access and lack of control is that the DSI older adults primarily need interaction and alignment with their environment. Although they are well aware of the existence of the environment, without interaction, information, and alignment, the DSI older adults are unable to function as a social being nor as a person undertaking daily activities. Interactions during daily care, receiving information, having conversations about daily care and on daily LTC circumstances, might enable the DSI older adults to connect and live their lives as human beings, in connection with others. This form of support requires person-centered communication attitudes and LTC professionals’ skills, especially of the nurses who provide daily care.

### Strengths and limitations

This study contributes to the limited body of qualitative data regarding the problems of DSI older adults in LTC. As the problems were collected at an individual level between older adult and a familiar nurse, our study provides in-depth insights into the personal and actual problems, which could not have been achieved via previous population-based data collection using inventories or observation scales. However, the involvement of a familiar nurse may have introduced bias due to DSI older adults’ feelings of dependency of the nurse for their care; they may therefore have had difficulties expressing care-related problems. In a separate study, describing nurses’ changing perceptions in the course of the intervention period, nurses observed an initial reluctance among the DSI older adults to share their problems [32]. However, they also observed that this reluctance disappeared when nurses invited the DSI older adults to jointly search for and think about alternative solutions. The findings in this study support nurses’ observations that the older adults overcame their initial reluctance and felt free to express care-related problems, as the categories care delivery (theme participation) and lack of control on the support of the nurses (theme CIPS) only reflect problems related to the behaviors of professional care workers. Nevertheless, our finding that all of the problems identified by the older adults focused solely on LTC situations and that none of the older adults shared problems related to their family or private life, may suggest that the older adults did not completely feel free. This may be explained by their unfamiliarity with the role of the nurses as a partner in discussing problems, and by their attempts to uphold their socially valued identity as a family member [33]. Another limitation in this study was that the interviewing approach of the SMP-DSI did not allow the inclusion of DSI older adults with severe cognitive problems, whereas recent research shows that an increasing number of DSI older adults in LTC suffer from severe cognitive impairment [2, 6]. Although our findings cannot be generalized to a population with severe cognitive problems and DSI, this first qualitative insight into the problems collected among DSI older adults who were able to articulate their problems, might contribute to the knowledge on those DSI older adults who are not able to clearly express their problems and needs. Further work is required to develop tools that enable observation and identification of the problems of the latter target group.

In conclusion, this study is one of the first to offer insights into the problems perceived by DSI older adults in LTC. These problems show great similarities with the problems experienced by deafblind individuals towards access in communication, information and mobility, but with the addition of existential concerns and lack of control for the DSI older adults. The findings highlight the importance of identifying the problems among this fast-growing group of DSI older adults in LTC and urges LTC facilities and carers to reconsider what they offer as usual care. To address the participation and CIPS problems of DSI older adults, LTC professionals and researchers need to develop and test programs providing autonomy based and person-centered participation support such as, for example, the SMP-DSI program.

## Supporting information

S1 Table Semi-Structured Intervention Diary for Nurses. (DOCX)

S1 Fig CONSORT Flow Diagram of cluster Randomized Controlled Trial. (PDF)

S1 File Approval Medical Ethical Committee. (PDF)

S2 File Main points Ethical Approval. (DOCX)
