# Supplementary material for: Problems identified by dual sensory impaired older adults in long-term care when using a self-management program: A qualitative study
Source: PLoS One. 2017 Mar 21;12(3):e0173601. doi: 10.1371/journal.pone.0173601 (PMC5360251; doi:10.1371/journal.pone.0173601)
Supplement: S1 Table — (DOCX) [file pone.0173601.s001.docx]

**S1 Table. Semi-Structured Intervention Diary for Nurses**

| Name nurse:  Code older adult:  Please fill in the diary during or directly after you have spoken to your client.  Note the answers (quotes) and reactions of the older adult per step. | |
| --- | --- |
|  |  |
| \| **STEPS** \| **ANSWERS AND REACTIONS – QUOTES** \| \| --- \| --- \| \| **Step 1**  Identification of problem or wish  *Would you like to do something about it?* \|  \| \| **Step 2**  Collecting Alternatives  *What could you do about it?*  *Are there other options?* \|  \| \| **Step 3**  Choice and Planning  *How do you think you will manage this?* \|  \| \| **Step 4**  Execution \|  \| \| **Step 5**  Reflection  *What was the result?*  *What are you happy about: about what you could do on your own?*  *What would you do differently the next time?* \|  \| |  |
