# Supplementary figures and images for: Problems identified by dual sensory impaired older adults in long-term care when using a self-management program: A qualitative study
Source: PLoS One. 2017 Mar 21;12(3):e0173601. doi: 10.1371/journal.pone.0173601 (PMC5360251; doi:10.1371/journal.pone.0173601)

**S1 Figure. CONSORT FLOW DIAGRAM OF THE cRCT**

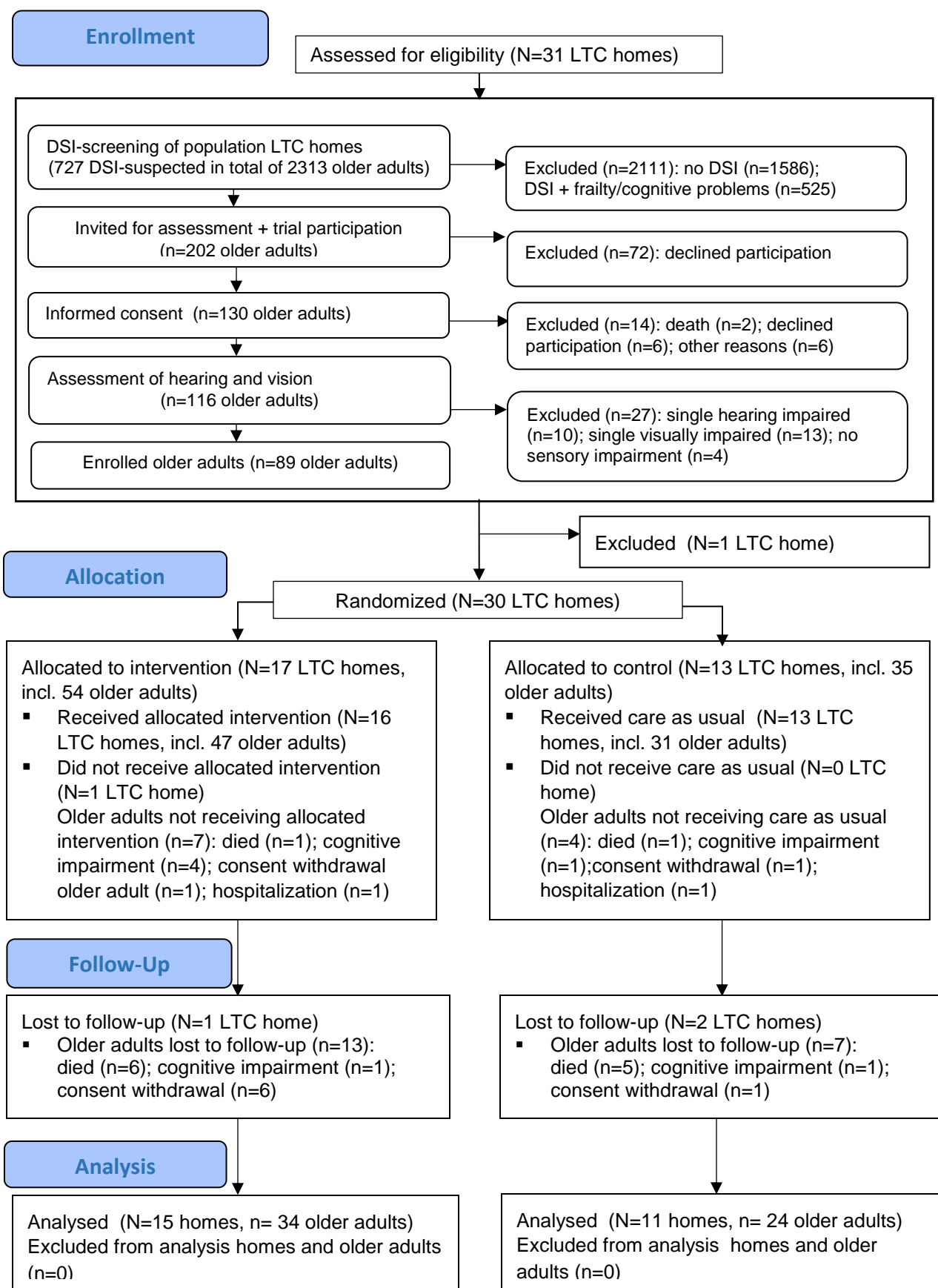

N = number LTC homes; n = number older adults.

Supplement: S1 Fig — (PDF) [file pone.0173601.s002.pdf]
