# Supplementary material for: Problems identified by dual sensory impaired older adults in long-term care when using a self-management program: A qualitative study
Source: PLoS One. 2017 Mar 21;12(3):e0173601. doi: 10.1371/journal.pone.0173601 (PMC5360251; doi:10.1371/journal.pone.0173601)
Supplement: S1 File — (PDF) [file pone.0173601.s003.pdf]

Nurse-Supported Self-Management for Dual Sensory Impaired Older Adults in Long-Term Care: Effect and Process Evaluation of a Cluster Randomized Controlled Trial

---

Documents on the application to and approval of the ethics committee for the clinical trial as described in the study protocol (1). The study protocol has been included as supplementary information in the submission to Plos One.

Scan of 3 documents:

1. First document, one page:

Approval of the Commissie Mensgebonden Onderzoek Arnhem-Nijmegen (Committee Research on Humans region Arnhem-Nijmegen) in the Netherlands.

Translation of the text of the approval-letter:
Title: Self-Management for deafblind older adults and their informal caretakers: an effective approach?
Hereby I inform you that the CMO region Arnhem-Nijmegen gives approval for above-mentioned research to the Board of Kalorama which declares that he research can be performed on location.

2. Second document, 12 pages: B 1
ABR-Formulier voor medisch-ethische beoordeling en registratie (Application form for medical-ethical assessement and registration of the Committee Research on Humans).
Page 11 and 12 give information in English.

3. Third document, 12 pages: C 1
Research protocol in which the clinical trial was embedded. This protocol is in Dutch, and corresponds with the information given in the ABR-form.

---

1. Roets-Merken LM, Graff MJ, Zuidema SU, Hermsen PG, Teerenstra S, Kempen GI, et al. Effectiveness of a self-management program for dual sensory impaired seniors in aged care settings: study protocol for a cluster randomized controlled trial. Trials. 2013;14:321.

2 5 JUNI 2009

6552.

Commissie Mensgebonden Onderzoek
Regio Arnhem – Nijmegen

Canisius Wilhelmina Ziekenhuis
UMC St Radboud
Ziekenhuis Rijnstate/AlysisZorggroep
St. Maartenskliniek

Raad van Bestuur van
Stichting Kalorama
Nieuwe Holleweg 12
6573 DX Beek-Ubbergen

Ons Kenmerk
CD/CMO 1231

Datum
22 juni 2009

Titel: Zelfmanagement voor doofblinde ouderen en hun mantelzorgers: een effectieve aanpak?
CMO dossiernr: 2008/314
ABR nr.: NL26192.091.08

Bij dezen laat ik u weten dat de CMO regio Arnhem-Nijmegen een positief oordeel over bovengenoemd onderzoek zal geven nadat de Raad van Bestuur van Kalorama verklaard heeft dat het onderzoek lokaal uitvoerbaar is.

Met vriendelijke groeten,

Namens de CMO regio Arnhem-Nijmegen

Mr. dr. F. van Agt

Cc: Stichting Kalorama, L.M. Roets-Merken, Nieuwe Holleweg 12, 6573 DX Beek-Ubbergen

Postadres
CMO Regio Arnhem-Nijmegen
p/a UMC St Radboud
Huispost / Route 578
Postbus 9101
6500 HB Nijmegen

Bezoekadres
UMC St Radboud
Th. Craanenlaan 6
6525 GH Nijmegen

Tel. 024-3613154
Fax 024-3610148
E-mail cmo@medzaken.umcn.nl
Internet www.umcn.nl

B 1

Sluiten

Formulier voor medisch-ethische beoordeling en registratie

ABR-formulier, versie juni 2007

Onderzoeksdossiernummer

ABR Nummer	**26192**	Status	**Definitief**
Versie	**01**	Status per	**23-12-2008**
Jaar	**08**		
Dossiernummer	**NL26192.091.08**		

A. Sectie - Openbaar maken gegevens medisch wetenschappelijk onderzoek

A1. De CCMO streeft naar meer transparantie over (de beoordeling van) medischwetenschappelijk onderzoek met mensen, geslachtscellen, embryo's en foetussen in utero. De verrichter (opdrachtgever/sponsor) kan hier aangeven toestemming te verlenen voor de openbaarmaking van de antwoorden op de vragen gemarkeerd met een wereldbol en de samenvatting bij dit formulier via de CCMO-website, dan wel de openbaarmaking te weigeren:

- (•) de verrichter verleent de CCMO toestemming voor de bovengenoemde openbaarmaking
- ( ) de verrichter verleent de CCMO géén toestemming voor de bovengenoemde openbaarmaking

B. Sectie - Administratief

B1. Betreft het onderzoek met geneesmiddelen (inclusief gentherapie, somatische celtherapie, vaccinonderzoek, GGO's, zie verdere toelichting) als bedoeld in de Wet medisch-wetenschappelijk onderzoek met mensen (WMO)?

- ( ) ja
- (•) nee

B2. Houdt het onderzoek verband met een eerder door een erkende METC of door de CCMO beoordeelde studie of is het onderzoek reeds eerder bij een erkende METC ter beoordeling voorgelegd?

- ( ) ja, het onderzoek houdt verband met - of is het vervolg op - een eerder beoordeelde studie
- ( ) ja, het onderzoek is eerder ter beoordeling aan een erkende METC of de CCMO voorgelegd (stuur kopie besluit mee)
- (•) nee

B3. Is het protocol reeds door een andere instantie op zijn wetenschappelijke aspecten beoordeeld?

- ( ) ja (stuur kopie van beoordeling mee)
- (•) nee

B4. Is het protocol (nog) in een ander openbaar trial register geregistreerd?

- ( ) ja
- (•) nee

B5. Naam indiener/contactpersoon voor de oordelende toetsingscommissie

Achternaam indiener/contactpersoon	Roets-Merken
Titel en voorletters	Mw. G.M.
Tussenvoegsel	
Type Organisatie/Bedrijf	Overig
Organisatie/Bedrijf	Anders, nl.

Voer naam Organisatie/Bedrijf in	Stichting Kalorama
Afdeling	
Intern adres	
Adres	Nieuwe Holleweg 12
Postcode en plaats	6573 DX Beek-Ubbergen
Land	Nederland
Telefoon	024-6847777
Fax	
E-mail	l.roets@kalorama.nl

B6. Is de indiener werkzaam bij de opdrachtgever/sponsor (verrichter) van het onderzoek?

(•) Ja ( ) Nee

C. Sectie - Onderzoek

C1. Volledige titel van het onderzoek

C1a. In het Engels

Self-management for dual sensory impaired older people and their significant others: an effective approach?

C1b. In het Nederlands

Zelfmanagement voor doofblinde ouderen en hun mantelzorgers: een effectieve aanpak?

C2. Verkorte titel van het onderzoek/acroniem

C2a. In het Engels

Self-management and dual sensory loss

C2b. In het Nederlands (Let op: deze korte titel wordt vermeld binnen ToetsingOnline)

Zelfmanagement bij doofblindheid

C3. Trefwoorden (maximaal 4, plaats elk trefwoord op een aparte regel)

C3a. In het Engels

- dual sensory impairment
- psychosocial intervention
- elderly
- signifant other

C3b. In het Nederlands

- zintuiglijke beperking
- psychosociale interventie
- ouderen
- mantelzorgers

C4. Beschrijf het belang van het onderzoek en de beoogde toepassing van de resultaten (verwijs eventueel naar de relevante pagina's in het protocol).

Zie protocol pagina 4, hoofdstuk 11.

C5. Wat is de hoofdvraagstelling van het onderzoek?

Wat zijn de effecten van de Zelfmanagement Interventie Gids doofblinden op de autonomie van de doofblinde oudere, het competentiegevoel van de mantelzorger en de arbeidssatisfactie van de professionele verzorgende en begeleider?

C6. Betreft het onderzoek een multicenter-onderzoek?

( ) nee
(•) ja - alleen in Nederland
( ) ja - internationaal binnen de Europese Unie
( ) ja - internationaal ook buiten de Europese Unie

C7. Is er bij multicenter-onderzoek sprake van een coördinerend onderzoeker?

* (•) ja namelijk — Naam onderzoeker: L. Roets
( ) nee

C8. Wie is/zijn medisch verantwoordelijk voor de proefpersonen die deelnemen aan het onderzoek

Geen artsen betrokken bij dit psychosociaal interventieonderzoek.

C9. In welk centrum/welke centra (incl. huisartsenpraktijken) in Nederland wordt het onderzoek uitgevoerd? Geef per centrum op: aantal proefpersonen, naam hoofdonderzoeker, naam onafhankelijk arts.

Centrum Informatie

Centrum	Proefpersonen	Hoofdonderzoeker	Onafhankelijk arts
Amsterdam Thuiszorg	12	L. Roets	niet bij betrokken
Bartimeus, hulpverlening voor slechtzienden	12	L. Roets	niet bij betrokken
Icare Thuiszorg	12	L. Roets	niet bij betrokken
Professor Heymansstichting, Friesland	16	L. Roets	niet bij betrokken
Sensis (lokatie de Vlasborch Vught)	10	L. Roets	niet bij betrokken
Stichting De Wever, Tilburg	10	L.Roets	niet bij betrokken
Stichting Vitalis	10	L. Roets	niet bij betrokken
Visio (lokaties Zeeland, Noord- en Zuid-Holland)	20	L. Roets	niet bij betrokken
Zorgcentrum Goverwelle, Gouda	10	L. Roets	niet bij betrokken

C10. Betreft het onderzoek met:

- [x] mensen
- [ ] geslachtscellen
- [ ] (rest-)embryo's
- [ ] foetussen in utero

C11. Beoogd totaal aantal proefpersonen/(rest)embryo's/foetussen in utero:

C11a. In Nederland
112

C12. Op welke pagina van het onderzoeksprotocol wordt een onderbouwing gegeven van het aantal proefpersonen/(rest-)embryo's/foetussen in utero?

pagina 7

C13. Onderzoeksgebied

- [ ] etiologie
- [ ] organisatorisch/zorgonderzoek
- [ ] diagnostiek
- [ ] preventie
- [ ] therapie
- [ ] veiligheid
- [ ] werkzaamheid
- [ ] farmacokinetiek
- [ ] farmacodynamiek
- [ ] bio-equivalentie
- [ ] dosis-respons
- [ ] farmacogenomics
- [ ] farmaco-economie

☑ anders

namelijk psycho-sociale interventie

C14. Type onderzoek

- ○ observationeel onderzoek zonder invasieve metingen
- ○ observationeel onderzoek met invasieve metingen
- ◉ interventie-onderzoek

C15. In welke fase kan het onderzoek worden ingedeeld?

- ○ fase I (a)
- ○ fase II (b)
- ○ fase III (c)
- ○ fase IV (d)
- ○ overige onderzoeken waarbij geneesmiddelen worden toegepast (e)
- ◉ niet van toepassing

C18. Is er sprake van (nog) een andere interventie dan met geneesmiddelen zoals bedoeld in de Wet Medisch Wetenschappelijk Onderzoek met Mensen (zie toelichting)?

- ◉ Ja
- ○ Nee

C18a. ☐ Medisch hulpmiddel, namelijk
C18b. ☐ Operatie, namelijk
C18c. ☐ Psychosociale interventie, namelijk
C18d. ☐ Voeding(stoffen), namelijk
C18e. ☐ Bewegingstherapie, namelijk
C18f. ☐ interventie met radioactieve straling, namelijk
C18g. ☐ Blootstellingsonderzoek (bijv pesticidenonderzoek), namelijk
C18h. ☑ andere interventie , namelijk

In het Engels: psychosocial intervention
In het Nederlands: psychosociale interventie

C19. Is/zijn er (een) controlegroep (en)?

- ◉ ja
- ○ nee
- ☐ ja, geneesmiddel
- ☐ ja, placebo
- ☑ ja, geen inteventie
- ☐ ja, anders

C20. Betreft het een gerandomiseerd onderzoek?

- ◉ ja
- ○ nee

namelijk

- ☑ open
- ☐ enkelblind
- ☐ dubbelblind
- ☐ parallel
- ☐ cross-over
- ☐ anders

C21. Op welke klasse(n) van aandoeningen heeft het onderzoek betrekking (maximaal 3)

- [ ] hartaandoeningen
- [ ] congenitale, familiaire en genetische aandoeningen
- [ ] bloed- en lymfestelsel aandoeningen
- [ ] zenuwstelsel aandoeningen
- [x] oogaandoeningen
  visusstoornissen
- [x] evenwichtsorgaan- en ooraandoeningen
  gehoorstoornissen
- [ ] ademhalingsstelsel-, thorax- en mediastinumaandoeningen
- [ ] maagdarmstelselaandoeningen
- [ ] nier- en urinewegaandoeningen
- [ ] huid- en onderhuidaandoeningen
- [ ] skeletspierstelsel- en bindweefselaandoeningen
- [ ] endocriene aandoeningen
- [ ] voedingsstoornissen en metabole ziekten
- [ ] infecties en parasitaire aandoeningen
- [ ] letsels, intoxicaties en verrichtingscomplicaties
- [ ] neoplasmata, benigne, maligne en niet-gespecificeerd (incl cysten en poliepen)
- [ ] chirurgische en medische verrichtingen
- [ ] bloedvataandoeningen
- [ ] algemene aandoeningen en aandoeningen op de plek van toediening
- [ ] zwangerschap, perinatale periode en puerperium
- [ ] sociale omstandigheden
- [ ] immuunsysteemaandoeningen
- [ ] lever- en galaandoeningen
- [ ] voortplantingsstelsel- en borstaandoeningen
- [ ] psychische stoornissen
- [ ] overig, namelijk

C22.Geef twee synoniemen voor de aandoening die bestudeerd wordt, waarvan tenminste één lekenterm.

In het Engels:	dual sensory loss
In het Nederlands:	dubbele zintuiglijke beperking

C23. Beoogde start- en einddatum van het onderzoek

C23a. Start Datum (dd-mm-jjjj)	01-02-2009
C23b. Eind Datum (dd-mm-jjjj)	01-01-2012

D. Sectie - Proefpersonen

D1. Is er een proefpersonenverzekering conform de WMO-eisen afgesloten of wordt aan de oordelende toetsingscommissie ontheffing gevraagd?

- ( ) proefpersonenverzekering is afgesloten bij verzekeringsmaatschappij
- (•) ontheffing van de verzekering wordt gevraagd
- ( ) niet van toepassing - onderzoek valt onder de Embryowet en niet onder de WMO

D2. Gezonde proefpersonen en/of patiënten

- [ ] Gezonde proefpersonen
- [x] Patiënten

Aantal 112

D3. Welke specifieke populaties worden voor deelname aan het onderzoek benaderd?

Personen van 55 jaar of ouder die slechthorend en slechtziend geworden zijn. Thuiswonend of in een verzorgingshuis.

D4. Voornaamste inclusiecriteria

D4a. In het Engels
- Participants aged 55 or over
- Participants with a dual sensory loss
- Community-dwelling or living in a home for the elderly

D4b. In het Nederlands
- personen van 55 jaar of ouder
- slechtziende en slechthorende ouderen
- thuiswonend of in een verzorginghuis verblijvend

D5. Voornaamste exclusiecriteria

D5a. In het Engels
- prelingual deaf participants
- participants with a dual sensory loss acquired before the age of 55
- participants living in a nursing home

D5b. In het Nederlands
- prelinguaal dove personen
- personen met een vroeg verworven dooflblindheid (= voor leeftijd van 55 jaar)
- in een verpleeghuis verblijvend

D6. Bij welke categorie proefpersonen wordt het onderzoek uitgevoerd (meerdere antwoorden mogelijk)

- [x] 18 jaar of ouder en wilsbekwaam **(ga naar vraag D10)**
- [ ] 18 jaar of ouder en wilsonbekwaam **(ga naar vraag D7)**
- [ ] 12 t/m 17 jaar en in staat tot het geven van geïnformeerde toestemming **(ga naar vraag D8)**
- [ ] 12 t/m 17 jaar en niet in staat tot het geven van geïnformeerde toestemming (wilsonbekwaam) **(ga naar vraag D7)**
- [ ] jonger dan 12 jaar **(ga naar vraag D8)**

D10. Verkeren (sommige) proefpersonen in een afhankelijkheidssituatie ten opzichte van de onderzoeker of degene die de deelnemers werft? (lees de toelichting voor voorbeelden wanneer er sprake kan zijn van een afhankelijkheidssituatie)

- ( ) ja
- (•) nee

D11. Waaruit bestaat de vergoeding voor de proefpersonen?

- [x] geen vergoeding
- [ ] reiskosten
- [ ] financiële vergoeding (in Euro's)
- [ ] andere vergoeding

D12. Is deze vergoeding afhankelijk van bepaalde voorwaarden, bijvoorbeeld het voltooien van (een deel van) het onderzoek?

- ( ) ja (motiveer)
- ( ) nee
- (•) niet van toepassing

E. Sectie - Voor- en nadelen

E1. Wordt er bij dit onderzoek een rechtstreeks therapeutisch effect beoogd bij de

proefpersonen / patiënten?

( ) ja (therapeutisch onderzoek)
(•) nee (niet-therapeutisch onderzoek)

E1b. Zo nee, kan deelname op een andere manier ten goede komen aan de proefpersoon?

(•) ja (motiveer)
( ) nee

namelijk toename autonomie en welzijn

E2. Waaruit bestaat de belasting van het onderzoek (en een eventueel daaraan voorafgaande keuring) voor de proefpersonen?

Tijdbeslag	per bezoek	anderhalf a twee uur
	totaal	10u programma + 3u vragenlijsten
	totale duur van de studie voor de individuele proefpersoon	max. 13 uur

E3. Worden de proefpersonen in verband met het onderzoek in het ziekenhuis opgenomen of wordt een opname verlengd?

( ) ja - het verblijf in het ziekenhuis/instituut wordt in verband met het onderzoek verlengd
( ) ja - ze worden voor het onderzoek in het ziekenhuis/instituut opgenomen
(•) nee

E4. Beschrijf in hoeverre proefpersonen worden onderworpen aan handelingen dan wel een gedragswijze krijgen opgelegd, zoals vragenlijst, interviews, lichamelijk/psychologisch onderzoek, ontzegging, dieet (voor invasieve ingrepen: zie vraag E6)

In deel 1: diepte-interview
In deel 2: vragenlijsten tijdens voor- en nameting, vaardigheden leren tijdens interventieprogramma

E5. Worden de proefpersonen getest op bepaalde aandoeningen/condities?

( ) ja (motiveer)
(•) nee

E6. Welke extra (invasieve) ingrepen (anders dan bij de standaard behandeling) moeten de proefpersonen in het kader van het onderzoek ondergaan:

[x] Niet van toepassing

E7. Welke maatregelen zijn genomen om (eventuele) risico's van de bijkomende ingrepen te vermijden en nadelige gevolgen te beperken?

n.v.t.

E8. Hoe is de spreiding van de ingrepen in de tijd, met name afname van bloed en ander lichaamsmateriaal (verwijs eventueel naar de flow chart in het protocol)?

n.v.t.

E9. Geef aan welke risico's er voor proefpersonen zijn verbonden aan deelname aan het onderzoek.

Tijdsinvestering van de proefpersonen.

E9a. Geef op grond van uw eigen afweging aan waarom het uitvoeren van het onderzoek, in het licht van de belasting en/of risico's die voor proefpersonen aan deelname verbonden zijn, gerechtvaardigd is?

Jarenlange ervaring met deze doelgroep laat zien dat de specifieke kwetsbaarheid van deze ouderen in de huidige zorg niet onderkend wordt terwijl de psychosociale gevolgen van de beperking groot zijn. Het is nodig dat de problematiek helder wordt geformuleerd, gebaseerd op controleerbaar en gedegen onderzoek, en dat er vervolgens een evidence based handreiking wordt geboden om de problemen voor te zijn c.q. beperkt te houden.

E10. Indien het onderzoek bij minderjarige en/of wilsonbekwame proefpersonen wordt uitgevoerd en geen direct therapeutisch effect wordt beoogd: waarom kunnen belasting en risico's als minimaal worden beschouwd? (Verwijs eventueel naar de relevante pagina's in het protocol.)

☑ niet van toepassing

E11. Kan de eventuele therapie na beëindiging van het onderzoek worden voortgezet?

○ ja (motiveer)

○ nee (motiveer)

◉ niet van toepassing

E12. Heeft deelname aan het onderzoek voor de proefpersoon tot gevolg dat van de standaardbehandeling of -diagnostiek kan worden afgeweken of deze kan worden uitgesteld?

○ ja

○ nee

◉ niet van toepassing

F. Sectie - Informatie en privacy

F1. Hoe worden de proefpersonen geworven en door wie (onderzoeker, behandelend arts, andere persoon) wordt de proefpersoon/wettelijke vertegenwoordiger geïnformeerd en om toestemming gevraagd?

Kandidaten worden via de Voorziening Betaalde Begeleiders voor Doofblinden (voor diepte-interviews), via Regionale Instellingen voor Blinden en Slechtzienden, verzorgingshuizen en thuiszorgorganisaties geworven. Medewerkers van deze organisaties beschikken over informatiefolders en leggen de eerste contacten. Vervolgens informeert de onderzoeker de kandidaten en deze is ook verantwoordelijk voor het verkrijgen van de toestemmingen voor deelname en opvragen van audiologische en visuele metingen.

F2. Hoeveel bedenktijd krijgen de proefpersonen/wettelijke vertegenwoordigers om te beslissen over deelname?

twee weken

F3. Wordt de huisarts, behandelend specialist en/of apotheker van de proefpersoon geïnformeerd over diens deelname aan het onderzoek?

◉ ja (de proefpersoon dient hiervoor toestemming te geven)

○ nee

F4. Worden persoonsgegevens gecodeerd?

◉ ja

○ nee

F4a. Zo ja, hoe is deze codering opgebouwd?

De codering bestaat uit een combinatie van een letter en vier cijfers: het eerste cijfer als aanduiding voor thuis/verzorgingshuiswonend, de overige drie voor de proefpersoon. De letters P, M en V staan voor participant, mantelzorger en verzorgende/begeleider. De vertaling wordt afgesloten bewaard.

F4b. Wie heeft toegang tot de sleutel van deze code?

Onderzoeker, aan het eind van het onderzoek

F4c. Wie hebben toegang tot de brondocumenten en eventuele andere tot de persoon herleidbare gegevens?

Onderzoeker

F5. Hoe wordt het lichaamsmateriaal gedurende het onderzoek bewaard?

- ○ in tot de proefpersoon herleidbare vorm
- ○ in niet tot de proefpersoon herleidbare vorm (volledig geanonimiseerd)
- ◉ niet van toepassing

F6. Wordt afgenomen lichaamsmateriaal na afloop van het onderzoek vernietigd?

- ○ ja
- ○ nee (motiveer)
- ◉ niet van toepassing

F7. Kunnen proefpersonen na afloop van het onderzoek opnieuw benaderd worden (bijvoorbeeld voor nader onderzoek of follow-up)?

- ○ ja
- ◉ nee

G. Sectie - Financieel

G1. Door welke geldstroom wordt het onderzoek gefinancierd?

- ☐ eerste geldstroom (Geld van Ministerie van OC&W aan universiteiten)
- ☐ tweede geldstroom (NWO of KNAW), namelijk:
- ☑ derde geldstroom (anders dan 1e of 2e geldstroom, zoals collectebusfondsen, Europese Unie, vakministeries of bedrijven), namelijk:
  Stiching St Joannes de Deo en Moeder Catharinafonds

G2. Wordt het onderzoek (mede) gefinancierd door de industrie/bedrijven?

- ○ ja - door de industrie/bedrijf zoals is opgegeven bij sectie B (opdrachtgever van het onderzoek)
- ○ ja - (ook) door andere industrie/bedrijven dan de opdrachtgever
- ◉ nee

G3. Ontvangt de arts/onderzoeker persoonlijk (afgezien van zijn/haar normale salaris) een vergoeding voor de uitvoering van het onderhavige onderzoek?

- ○ ja
- ◉ nee

G4. Heeft/hebben de onderzoeker(s) gedurende de afgelopen vijf jaar op een of andere wijze een persoonlijke financiële relatie (gehad) met de verrichter/sponsor van het huidige onderzoek?

- ◉ ja (licht toe)
- ○ nee

G4a. Zo ja, geef aan welke relatie dit is of is geweest
Onderzoeker is in loondienst van de stichting Kalorama.

H. Sectie - Publicatiebeleid

H1. Zijn door de opdrachtgever/subsidiegever bepaalde voorwaarden gesteld aan het openbaar maken van de onderzoeksresultaten?

- ○ ja (licht toe)
- ◉ nee

I. Sectie - Indiening en beoordeling

I1. Selecteer de toetsingscommissie die het oordeel geeft in de zin van de WMO

Commissie
CMO regio Arnhem-Nijmegen (Nijmegen)

J. Sectie - Aanvullende opmerkingen

Aanvullende opmerkingen

Omtrent samenwerking met de onder C9 genoemde centra: de verwachting is dat zij deel zullen nemen. Formele afspraken worden later gemaakt.

K. Sectie - Samenvatting

Achtergrond van het onderzoek:

Uit prevalentieonderzoek (Vaal, 2007) blijkt dat 35 000 ouderen in Nederland een gecombineerde visuele en auditieve beperking hebben. De twee zintuiglijke functiebeperkingen versterken elkaar en veroorzaken een ernstige handicap die uniek is en doofblindheid genoemd wordt (DbI 2005). De getroffen ouderen ervaren problemen met de communicatie, de toegang tot informatie en mobiliteit. Zij voelen zich vaker geïsoleerd, eenzaam, depressief (Capella-Mc-Donall, 2005). Gemis aan informatie bedreigt het zelf keuzes kunnen maken en beslissingen nemen, en maakt de oudere persoon afhankelijk van anderen. Vertrouwde communicatiemethoden zijn onvoldoende toepasbaar waardoor de oudere persoon sociaal en emotioneel negatieve gevolgen ondervindt. Zij worden eerder opgenomen in instellingen (verpleeghuis bijvoorbeeld) (Jee e.a., 2005).

Het welzijn van kwetsbare ouderen kan verbeterd worden door zelfmanagementvaardigheden (Lorig e.a., 2005, Kempen, 2006). Tot op heden is er geen Nederlands onderzoek verricht onder doofblinde ouderen naar de gevolgen van doofblindheid noch naar mogelijke ondersteunende interventies zoals zelfmanagement.

Doel van het onderzoek:

Het doel van het onderzoek is het ontwikkelen en evalueren van de Zelfmanagement Interventie Gids doofblinden (ZIGdb) als middel om de communicatie en informatieverwerving tussen de oudere en zijn omgeving te verbeteren. De ZIGdb wordt geschikt gemaakt voor grootschalige verspreiding. Bij succes wordt implementatie in de reguliere zorg nagestreefd.

Onderzoeksopzet:

- Eerste fase: zelfmanagementinterventie ontwikkelen op basis van systematische literatuurstudies en diepte interviews (20 paren van doofblinde ouderen en hun mantelzorger)
- Tweede fase: een open randomised controlled trial (=112 paren van doofblinde ouderen en hun mantelzorger)

Onderzoekspopulatie:

Personen van 55 jaar en ouder met een op volwassen leeftijd verworven visuele en een auditieve beperking. Minstens een van beide beperkingen is pas verworven na het 53 ste levensjaar. De proefpersonen wonen thuis of verblijven in een verzorgingshuis en hebben contact met een mantelzorger. Exclusie geldt voor prelinguaal dove personen.

Interventie (indien van toepassing):

De interventie bestaat uit een individueel psychosociaal programma van 10 weken (5 x 2 u om de twee weken). De participanten worden getraind in communicatietechnieken, gespreksstrategieen, informatieverwerving en regievoering. De competenties waarop beroep wordt gedaan zijn kennisuitbreiding, bewustwording en vaardigheidsontwikkeling op drie levensgebieden van het dagelijks leven: communicatie, informatieverwerving en zelfstandig handelen. De mantelzorgers nemen deel aan twee van deze vijf sessies, gericht op gespreksvaardigheden en informatieverstrekking.

De interventie wordt geboden door verzorgenden van thuiszorg en verzorgingshuis die gecoacht worden door logopedisten/communicatiedeskundigen die gespecialiseerd zijn in de communicatie en omgang met doofblinde ouderen.

De controlegroepparticipanten en hun mantelzorgers ontvangen schriftelijke informatie in de vorm van een folder over verworven doofblindheid.

Primaire onderzoeksvariabelen/uitkomstmaten:

De beoogde primaire effecten zijn verbetering van het gevoel van autonomie van de doofblinde oudere en van competentiegevoelens bij de mantelzorgers.

Secundaire onderzoeksvariabelen/uitkomstmaten (indien van toepassing):
Secundaire uitkomstmaat voor de doofblinde ouderen: uitstel van opname in verpleeghuis.
Bij professionele verzorgers en begeleiders wordt als secundair effect een toename van de arbeidssatisfactie beoogd.

Omschrijving en inschatting van belasting en risico (indien van toepassing):

De tijdsbelasting van de deelnemers aan het diepteinterview bedraagt circa anderhalf uur per persoon, en van de mantelzorger een uur.
De totale tijdsbelasting van de interventiegroep is circa 13 uur (10 uur interventie en 2x 70 minuten mondeling vragenlijsten beantwoorden) per persoon, en voor de mantelzorger circa 2 1/2 uur (2 uur interventie en 2x 10 minuten vragenlijsten). De belasting van controlegroep is 2 x70 minuten, voor de mantelzorger 2 x 10 minuten.
Gezien de mobiliteitsproblemen van de doelgroep, vinden de interventies plaats in de woonomgeving van iedere proefpersoon.

K2.Engelse Samenvatting

Background of the study:
In the Netherlands the calculation of the prevalences of dual sensory loss (Vaal, 2007) estimated that there are 35 000 elderly persons suffering from this disease. The combination of the two impairments cause a severe handicap that is unique and is called deafblindness (DbI 2005). The affected persons are experiencing problems in communication, access to information and in mobility. Any degree of loss of sight or hearing can result in loneliness, depression and isolation (Capella-Mc-Donall, 2005). Lack of information removes the ability to make informed decisions and also threatens independence. The loss of conventional methods of communication can have devastating social and emotional consequences. The loss of mobility can mean the loss of independence and daily living skills. There is an augmented risk that deafblind elderly are transitioned from independent community living to institutionalized care (Jee, 2005).
The well being of vulnarable elderly can be increased by self management abilities (Lorig e.a., 2005, Kempen, 2006). Untill now there is no Dutch study done among deafblind elderly to search the consequences of deafblindness nor to the potential supportive interventions like self management.

Objective of the study:
The aim is to develop and to evaluate a self-management intervention guide for elderly with a dual sensory impairment. This guide is a tool meant to improve the communication and the acces to information. The guide will be made available for large-scale distribution. If the results proofed to be successful, implementation in regular care will be aimed.

Study design:
- Part 1: development of a self-management intervention based on findings of systematic studies of the literature and in depth interviews (20 couples of elderly with a dual sensory impairment and their significant other
-Part 2: an open randomized controlled trial (= 112 couples of elderly with a dual sensory impairment and their significant other)

Study population:
Persons of 55 years and older with a visual and hearing loss, both acquired in adulthood. At least one of these impairments has been acquired after the age of 53. The participants are community-dwelling or live in a home for the elderly. All participants have contact with a significant other. Prelingual deaf persons are excluded from participation.

Intervention (if applicable):
The intervention program consists of communication techniques, conversation strategies, acquiring access to information, maintenance of autonomy and grip on life. The intervention program that is offered to the significant others consists of communication techniques and giving information. De competentions that are involved are knowledge, awareness and strategies.
The intervention is offered by professional carers who are previously have been trained by communicationspecialists/speech therapists specialized in communication with elderly with a dual sensory impairment.

The participants of the controllgroup and their significant others receive written information: a leaflet about acquired dual sensory loss.

Primary study parameters/outcome of the study:
The primary outcome of the study is the sense of autonoy for the elder person with the dual sensory impairment, and feelings of competence for the significant other.

Secundary study parameters/outcome of the study (if applicable):
Secundary outcome for the eldelry: delay of institutionalization.
Secundary outcome for the professional carers: increasement of worksatisfaction

Nature and extent of the burden and risks associated with participation, benefit and group relatedness (if applicable):
The burden for all participants is the time they have to spend.
Time burden for the participants to the in depth interview is 1 1/2 hr pro deafblind person, and 1 hr pro significant other.
The intervention group needs to spend 13 hrs pro participant, their significant other spend 2 1/2hrs. The controll group spends 2 x 70 minits, the significant other 2 x 10 minits.
Due to the mobility problems of the dual sensory impaired participants, all interventions and interviews will take place in the living environment of these participants.

ONDERTEKENING

De verrichter en indiener verklaren hierbij:
a. het formulier (en samenvatting) volledig en naar waarheid te hebben ingevuld;
b. de antwoorden op de vragen uit het ABR-formulier niet in strijd zijn met het bijbehorende onderzoeksdossier en onderzoekscontract

Naar waarheid getekend, door de verrichter (=opdrachtgever)

datum 23-12-08.

Handtekening
naam P. Kuiper
functie Raad van Bestuur Stichting Kalorama

door de indiener

datum 23-12-08

Handtekening
naam L. Roets-Merken
functie Wetenschappelijk medewerker Kalorama

Sluiten

Protocol C1

Stichting Joannes de Deo

Aanvraag subsidiëring onderzoek

1. Titel van het onderzoek
Zelfmanagement voor doofblinde ouderen en hun mantelzorgers: een effectieve aanpak?

2. Plaats van het onderzoek
Het onderzoek zal thuis en in het verzorgingshuis plaatsvinden.

3. Periode van het onderzoek
2008-2012

4. Projectleider
Prof. Dr. M. Vernooij-Dassen. Kalorama/ Afdelingen Verpleeghuisgeneeskunde en Kwaliteit van Zorg UMC St Radboud

5. Overige betrokkenen en beoogd uitvoerder
Mevr. G.M. Roets-Merken, onderzoeker Kalorama
Onderzoeksassistent, nog niet bekend
Begeleidingscommissie:
Dhr. P. Hermsen, adjunct directeur Kalorama
Drs. S. U. Zuidema, verpleeghuisarts-onderzoeker Kalorama/ Verpleeghuisgeneeskunde UMC St Radboud
Prof. Dr. G.I. Kempen. Gezondheidswetenschappen Universiteit Maastricht
Drs. M. Graff, onderzoeker/ergotherapeut UMC St Radboud

6. Onderzoekslijnen van de projectleider onder vermelding van subsidiegever
Dementie onderzoek. Subsidiegevers o.a. ZonMw, Europese Unie, Alzheimer Nederland, Provincie Gelderland
Onderzoek palliatieve zorg: KWF Kankerbestrijding
Onderzoek organisatie van zorg en professioneel gedrag: Stichting Beroepsopleiding Huisartsen Nijmegen

7. Projectinbedding
Het project is ingebed in de Stichting Kalorama en in het UMC St Radboud. Het Centrum voor Doofblinden van Kalorama is een landelijk expertisecentrum in behandeling en begeleiding van doofblinden. Binnen het Radboud UMC is het onderzoek ingebed in het onderzoeksinstituut *Nijmegen Centre for Evidence-Based Practice (NCEBP)*. Het onderzoek binnen NCEBP, zowel fundamenteel als toegepast, richt zich op het ontwikkelen en evalueren van effectieve en efficiënte patiëntenzorg en op de implementatie hiervan. Het NCEBP is een breed samenwerkingsverband waarin geneeskunde, ethiek, epidemiologie, sociale wetenschappen, paramedische wetenschappen en verplegingswetenschappen samenwerken. Het onderzoek heeft onder meer betrekking op geriatrie en chronische ziekten en ontwikkelt en evalueert instrumenten voor verbetering van kwaliteit van zorg en strategieën om het gedrag van professionals te veranderen. Bij het onderzoek zijn twee afdelingen van NCEBP betrokken, Eerstelijns geneeskunde (Prof. Dr. C. van Weel) waaronder verpleeghuisgeneeskunde (Prof. Dr. Raymond Koopmans) en de Kwaliteit van Zorg (KWAZO) (Prof. Dr. R. Grol). KWAZO is een nationaal en internationaal erkend centre of

excellence.

Het voorliggend onderzoek betreft een promotieonderzoek van Mevr. G.M. Roets-Merken en wordt begeleid door Prof. Dr. M.Vernooij-Dassen hoogleraar Psychosociale aspecten van zorg voor kwetsbare ouderen, Joop Michels leerstoel; Prof. Dr. G.I. Kempen, hoogleraar sociale gerontologie en deskundige 'zelfmanagement'; Drs. S. Zuidema verpleeghuisarts/onderzoeker en Drs. M. Graff onderzoeker/ergotherapeut die dit jaar promoveert.

8. De beste recente publicaties van het projectteam

Vernooij-Dassen M J FJ, Osse H P, Schade E, Grol R P T M. Autonomy problems and needs for care in cancer patients in the palliative phase of the disease. J Pain Symptom Manage 2005; 30:264-70.

Osse H P, *Vernooij-Dassen* M J F, Schade E, Grol R P T M. A practical instrument to explore patientIs needs in palliative care: the Problems and Needs in Palliative Care questionnaire (PNPC-short version). Palliat Med 2007;21(5):391-9.

Graff MJL, Vernooij-Dassen, MJM, Thijssen M, Dekker J, Hoefnagels WHL, Olde Rikkert MGM. Effects of community occupational therapy on quality of life, mood and health status in dementia patients and their caregivers: a randomized controlled trial, Journals of Gerontology: Medical Science. J Gerontol A Biol Sci Med Sci. 2007; 62(9):1002-9.

Graff MJL, Vernooij-Dassen MJM, Thijssen M, Dekker J, Hoefnagels WHL, Olde Rikkert MGM. Community occupational therapy in patients with dementia and their caregivers: a randomized controlled trial. BMJ 2006;333: (7580):1196.

Vernooij-Dassen MJFJ, Groot MM, van den Berg J, Kuin A, van der Linden B, van Zuylen L, Crul BJP, Grol RPTM. Consultation in palliative care: the relevance of clarification of problems. Eur J of Cancer, 2007;436 (2): 316-22.

Jonkers C, Lamers F, Bosma H, Metsemakers J, *Kempen GIJM,* van Eijk J. Process evaluation of a minimal psychological intervention to reduce depression in chronically ill elderly persons. Patient Educ Couns. 2007; 68(3): 252-7.

Bouman A, Rossum E van, Ambergen T, *Kempen GIJM,* Knipschild P. Effects of a home visiting program for older people with poor health status: a randomized, clinical trial in the Netherlands. J Am Geriatr Soc, in press.

Zijlstra GAR, Haastregt JCM van, Rossum E van, Eijk JThM van, Yardley L, *Kempen GIJM.* Interventions to reduce fear of falling in community-living older people: a systematic review. J Am Geriatr Soc 2007; 55: 603-615.

Kempen GIJM, Yardley L, van Haasdregt JC, Zijstra GA, Beyer N, Hauer K, Todd C. The Short FES-I: a shortened version of the falls efficacy scale-international to assess fear of falling. Age Ageing 2008; 37(1): 45-50.

Zuidema SU, van Iersel MB, Koopmans RTCM, Verhey FRJ, Olde Rikkert MGM. Werkzaamheid en bijwijerkingen van antipsychotica voor neuropsychiatrische symptomen bij

dementie; een systematische review. Ned Tijdschr Geneesk 2006; 150: 1565-73.

Zuidema SU, Derksen E, Verhey FRJ, Koopmans RTCM. Prevalence of neuropsychiatric symptoms in a large sample of Dutch nursing home patients with dementia. Int J Geriatr Psychiatry 2007; 22: 632-638.

Bosman, I., *L. Roets* e.a.. Over Doofblindheid, communicatie en omgang. Agiel. 2006, Utrecht.

Wijngaarden, B., *L. Roets*, A. Witsiers. Ouderdomsdoofblindheid.Tijdschrift voor Verzorgenden. 2006;38:14-7.

9. Samenvatting

Inleiding

In Nederland hebben 35000 personen van 55 jaar en ouder een gecombineerde visuele en auditieve beperking. Dit heeft een enorme sociaal-emotionele impact op de persoon zelf, de mantelzorger en de professionele ondersteuner. De op latere leeftijd verworven doofblindheid leidt tot verlies aan autonomie en tot isolement. In de gebruikelijke hulpverlening wordt de communicatie met de oudere niet systematisch een centrale plaats gegeven. Er zijn binnen de institutionele zorg echter wel communicatiebenaderingen ontwikkeld.

Doelstelling

Het doel is het ontwikkelen en evalueren van de Zelfmanagement Interventie Gids doofblinden (ZIGdb) als middel om de communicatie en informatieverwerving tussen de oudere en zijn omgeving te verbeteren. De beoogde primaire effecten zijn verbetering van het gevoel van autonomie van de doofblinde oudere en van competentiegevoelens bij de mantelzorgers. Bij professionele verzorgers en begeleiders wordt als secundair effect een toename van de arbeidssatisfactie beoogd.
ZIGdb wordt beschikbaar gemaakt voor grootschalige verspreiding.

Vraagstelling

Wat zijn de effecten van de Zelfmanagement Interventie Gids doofblinden op de autonomie van de doofblinde oudere, het competentiegevoel van de mantelzorger en de arbeidssatisfactie van de professionele verzorgende en begeleider?

Methode

-Setting

Het onderzoek vindt plaats bij de oudere thuis en in het verzorgingshuis.

-Design

In de eerste fase wordt de zelfmanagement interventie voor doofblinde ouderen ontwikkeld op basis van systematische literatuurstudies en diepte interviews naar problemen en behoeften aan zorg bij doofblinde ouderen en hun mantelzorgers.
De effecten van ZIGdb op doofblinde ouderen, hun mantelzorgers en professionele verzorgenden en begeleiders worden onderzocht in een randomised controlled trial (n=112 paren van doofblinde ouderen en hun mantelzorgers). De interventie zal 1 jaar duren. Er zal in de experimentele groep een meting voorafgaande aan de interventie plaatsvinden en een na afloop van de interventie. In de controlegroep zal eveneens een voormeting plaatsvinden en een jaar later een nameting. Na afronding van de interventie zal de ZIGdb gereed worden gemaakt voor grootschalige verspreiding en verspreiding via Internet. Tevens zal een focusgroep-onderzoek onder uitvoerders van de interventie worden gehouden over factoren die interventie bevorderd dan wel belemmerd hebben. Hieruit zullen aanbevelingen voor implementatie worden afgeleid.

-Meetinstrumenten

De effecten worden vastgesteld met gevalideerde meetinstrumenten. Primair effect oudere doofblinde: autonomie (Autonomy Questionnaire). Primair effect mantelzorger:

competentiegevoelens (Sence of Competence Questionnaire). Secundaire effecten doofblinde oudere: depressie (CES-D), isolement (Isolement) en uitstel van opname in verpleeghuis (tijdstip opname). Secundair effect professionele verzorgende en begeleiders: arbeidssatisfactie (Arbeidssatisfactie).

Verwachte effecten

De verwachte effecten zijn verbetering van autonomie van de oudere met doofblindheid, verbeterde competentiegevoelens van de mantelzorgers en verbeterde arbeidssatisfactie van verzorgenden en begeleiders.

10. Samenvatting van de subsidieaanvraag voor leken

Bij 35000 ouderen in Nederland komt een gecombineerde visuele en auditieve beperking voor. Hierdoor worden zij vaak afhankelijk van hulp van anderen, waardoor zij de regie over hun leven in handen moeten leggen van hun mantelzorger of van hulpverlening. Doofblinde ouderen voelen zich vaker geïsoleerd, eenzaam, depressief en vertonen vaker claimend of apathisch gedrag. Ook raken zij eerder in een psychose (werkelijkheid vervormd waarnemen). Doofblinde ouderen worden eerder opgenomen in instellingen (verpleeghuis bijvoorbeeld), omdat daar een fysiek veilige omgeving geboden kan worden.
Zelfmanagement beoogt het verlies aan autonomie tegen te gaan door de oudere te ondersteunen op drie levensgebieden: a) het overzicht en grip krijgen op de behandeling en begeleiding die hem geboden wordt, b) het ontwikkelen en behouden van competenties om te kunnen blijven communiceren met zijn omgeving en deel te blijven uitmaken van die omgeving; c) het leren hanteren van emotionele veranderingen als gevolg van de beperkingen. Er zal een Zelfmanagement Interventie Gids doofblinden (ZIGdb) worden ontwikkeld waarin gepoogd wordt het verlies aan autonomie te beperken. In het onderzoek worden personen die aan het zelfmanagement programma deelnemen vergeleken met personen die niet aan dit programma deelnemen. Professionele verzorgenden en begeleiders worden opgeleid om het ZIGdb gedurende een jaar toe te passen bij oudere doofblinden en hun mantelzorgers thuis of in het verzorgingshuis. Bij 112 paren van doofblinde ouderen en hun mantelzorgers zal worden nagegaan of de autonomie verbetert. Daarnaast zal worden onderzocht of depressieve symptomen en isolement verminderen en opname in het verpleeghuis wordt uitgesteld. Tevens wordt onderzocht of het competentiegevoel bij mantelzorgers verbetert en de arbeidssatisfactie van verzorgenden en begeleiders toeneemt.
De verwachte effecten zijn verbetering van autonomie van de oudere met doofblindheid, verbeterde competentiegevoelens van de mantelzorgers en verbeterde arbeidssatisfactie van verzorgenden en begeleiders.

11. Belang van het onderzoek voor het bevorderen van het welzijn van doofblinde ouderen

Doofblindheid vormt een directe bedreiging voor de autonomie van de persoon en isoleert hem van zijn omgeving. Doofblindheid beperkt de persoon in zijn communiceren, zijn informatieverwerving en in zijn zelfstandig handelen.
Mantelzorgers voelen zich zwaar belast omdat doofblindheid een beroep doet op hun doorlopende ondersteuning. Tot nog toe is er geen onderzoek gedaan naar de behoeften van doofblinde ouderen en van hun mantelzorgers. Ook is er geen onderzoek gedaan naar vormen van ondersteuning die verbetering brengen in de autonomie van de doofblinde ouderen, competentiegevoelens van de mantelzorgers en arbeidssatisfactie van verzorgenden en begeleiders.
Het belang van dit onderzoek is kennis verwerven over de behoeften van doofblinde ouderen en hun mantelzorgers en kennis over de effecten van zelfmanagement op autonomie, competentiegevoelens van de mantelzorgers en arbeidssatisfactie van verzorgenden en begeleiders. Deze kennis heeft directe praktische waarde en voegt bij succes een belangrijk

instrument toe voor de verbetering van de zorg voor doofblinden en hun mantelzorgers. Het vervaardigen van de Zelfmanagement Interventie Gids waarin het programma wordt beschreven is een belangrijk middel voor verdere verspreiding van de interventie.

12. Doel van de studie

Het doel is een zelfmanagement programma te ontwikkelen en te evalueren dat oudere doofblinden beter in staat stelt om regie over zijn leven te blijven voeren (autonomie). Daarnaast is het doel om de mantelzorgers te helpen zich competenter te voelen. De meest gangbare definitie van doofblindheid is: een combinatie van een visuele en auditieve beperking waardoor de persoon, ondanks optimale visuele en auditieve hulpmiddelen, problemen ervaart in de mobiliteit, communicatie en informatieverwerving en zonder de hulp van anderen niet in staat is deel te nemen aan het maatschappelijk leven (Dbl, Deafblind International,2005).
Het zelfmanagement programma zal in een Interventie Gids worden vastgelegd. Dit maakt verspreiding op grote schaal mogelijk. De gids zal ook op het Internet worden geplaatst.

13. Experimentele benadering

Randomised controlled trial naar effecten van zelfmanagement op basis van de Zelfmanagement Interventie Gids doofblinden (ZIGdb) op doofblinde ouderen en hun mantelzorgers. De interventie (toepassing ZIGdb) zal 1 jaar duren. Er zal in de experimentele groep een meting voorafgaande aan de interventie plaatsvinden en een na afloop van de interventie. In de controlegroep zal eveneens een voormeting plaatsvinden en een jaar later een nameting. Doofblinde ouderen zullen ad random bij de interventiegroep dan wel de controlegroep worden ingedeeld. Verzorgenden en begeleiders die ouderen in de interventiegroep begeleiden zullen geen doofblinde ouderen begeleiden in de controlegroep. De controlegroep ontvangt de gangbare zorg.

14. Achtergrond en verricht voorwerk

Uit prevalentieonderzoek (Vaal, 2007) blijkt dat 35000 ouderen in Nederland een gecombineerde visuele en auditieve beperking hebben. De term doofblindheid heeft betrekking op mensen die zowel problemen hebben met horen als met zien. De ernst van de zintuiglijke beperkingen kan variëren. De twee zintuiglijke functiebeperkingen versterken elkaar en veroorzaken een ernstige handicap die uniek is en niet vergeleken kan worden met de optelsom van de twee enkelvoudige zintuiglijke beperkingen. Alle doofblinde mensen ervaren problemen met de communicatie, de toegang tot informatie en mobiliteit. Hun specifieke behoeftes aan ondersteuning om de gevolgen van de beperkingen op te vangen, variëren enorm. Dit houdt verband met de leeftijd, de oorzaak en de aard van de doofblindheid (Dbl, 2005).

Doofblinde ouderen voelen zich vaker geïsoleerd, eenzaam, depressief, vertonen vaker claimend of apathisch gedrag (Capella-Mc-Donall, 2005). Ook raken zij eerder in een psychose (werkelijkheid vervormd waarnemen). Doofblinde ouderen worden eerder opgenomen in instellingen (verpleeghuis bijvoorbeeld), omdat daar een fysiek veilige omgeving geboden kan worden (Jee e.a., 2005). De doofblinde oudere verliest daardoor de bekendheid van zijn oude omgeving, de zintuiglijke beperkingen verhinderen dat hij zelfstandigheid en contacten opbouwt in een nieuwe omgeving. Het welzijn van kwetsbare ouderen kan verbeterd worden door zelfmanagementvaardigheden (Lorig e.a., 2005, Kempen, 2006). Tot op heden is er geen Nederlands onderzoek verricht onder doofblinde ouderen naar de gevolgen van doofblindheid noch naar mogelijke ondersteunende interventies zoals zelfmanagement.

Kalorama heeft jarenlange ervaring in het ondersteunen van doofblinden, onder meer van doofblinde ouderen. De ondersteuningsprogramma's die ambulante begeleiders van Kalorama aan doofblinde ouderen thuis geven, en die gericht zijn op reactivering en regie terug geven, geven positieve resultaten op het gebied van autonomie. Het verricht voorwerk bestaat uit een combinatie van ruime werkervaring van de beoogde onderzoeker en ruime onderzoekservaring van de beoogde projectleider. De beoogde onderzoeker heeft meer dan 30 jaar ervaring in de doofblindenzorg, en was verantwoordelijk voor de ontwikkeling van behandelprogramma's voor doofblind geboren kinderen en voor de ontwikkeling van intra- en extramurale zorg voor doofblinde volwassenen. Specifieke expertisegebieden zijn: diagnostiek en ontwikkelen interventieprogramma's op gebied van communicatie en psychische ondersteuning en scholen en coachen van professionals in de doofblindenzorg. De projectleider heeft ruime ervaring in het uitvoeren en begeleiden van randomised controlled trials bij ouderen en hun mantelzorgers, in implementatie onderzoek en in kwalitatief onderzoek. Zij heeft veel ervaring in de ontwikkeling van interventies waarin communicatie centraal staat.

15. Hypothesen

1. De Zelfmanagement Interventie Gids doofblinden ZIGdb leidt tot een positievere ontwikkeling bij doofblinden in de interventiegroep in vergelijking de controlegroep wat betreft autonomie (primair effect) en depressie, isolement en uitstel van opname in het verpleeghuis (secundaire effecten).
2. ZIGdb leidt tot een positievere ontwikkeling in de interventiegroep in vergelijking de controlegroep van het competentiegevoel bij mantelzorgers (primair effect) en van arbeidssatisfactie van verzorgenden en begeleiders (secundair effect).

16. Vraagstelling

De vraagstellingen zijn:

1. Welke problemen en behoeften aan zorg hebben doofblinde ouderen en hun mantelzorgers?
2. Welke effecten zijn bekend van interventies bij doofblinde ouderen en hun mantelzorgers?
3. Welke zelfmanagementprogramma (ZIGdb) kan op basis van deze zorgbehoeften en aanwezige mogelijkheden worden ontwikkeld?
4. Wat zijn de effecten van ZIGdb, ten aanzien van autonomie en uitstel van opname in het verpleeghuis, isolement en depressie bij doofblinde ouderen?
5. Wat zijn de effecten van ZIGdb op de gevoelens van competentie van de mantelzorgers en de arbeidssatisfactie van verzorgenden en begeleiders?
6. Welke aanbevelingen voor implementatie in de hele sector kunnen uit het focusgroep onderzoek bij verzorgenden en begeleiders worden afgeleid?

17. Studieopzet

A1) Systematische literatuurstudie naar problemen en behoeften aan zorg bij doofblinde ouderen en hun mantelzorgers.
A2) Systematische literatuurstudie naar effecten van interventies gericht op zelfmanagement bij doofblinde ouderen.
A3) Interviewguide op basis van literatuurstudie. Diepte interviews naar problemen en behoeften aan zorg.
B) Maken van Zelfmanagement Interventie Gids doofblinden (ZIGdb) op basis van resultaten van A. De ZIGdb biedt materiaal voor een opleiding op maat voor professionele verzorgenden en begeleiders en zal in ieder geval elementen van een communicatiebenadering bevatten die gericht is op a) contact leggen van de oudere met

anderen en het overzicht en grip krijgen op de behandeling en begeleiding die hem geboden wordt, b) het ontwikkelen en behouden van competenties en van kansen tot dingen doen die de oudere zelf kiest, c) het leren hanteren van emotionele veranderingen als gevolg van de beperkingen. Dit alles geënt op de normen en rollen die bij de individuele oudere passen. Deze opleiding op maat moet de verzorgers hen in staat stellen zorg op maat te leveren aan de cliëntparen. Er zal een pilotonderzoek worden uitgevoerd naar de toepasbaarheid en effecten van ZIGdb.
C) Design: randomised controlled trial naar effecten van ZIGdb op doofblinde ouderen en hun mantelzorgers.
De participanten (paren van doofblinde ouderen en hun mantelzorgers) zullen worden geworven via verzorgingshuizen, via de hulpverlening aan blinden en slechtzienden en via de audiologische centra.

De poweranalyse is gebaseerd op de uitkomstmaat gevoel van competentie. Dit is de enige uitkomstmaat waarbij gevoeligheid voor verandering is vastgesteld. Het is een proxy benadering omdat de uitkomstmaat niet eerder voor mantelzorgers van doofblinden, maar van dementerenden is gebruikt (Graff e.a., 2006). Er is echter nog geen longitudinaal onderzoek gedaan bij doofblinde ouderen en daarom is voor deze optie gekozen. Randomisatie vindt plaats op het niveau van de verzorgende en begeleiders, en wordt gestratificeerd naar het type verzorgende/begeleider (werkzaam in de thuiszorg dan wel in een instelling). Voor gevoel van competentie bij de familie is bij een andere doelgroep (dementerenden) een effect gezien van 17 punten verbetering op een standaard deviatie van 14 (Graff e.a., 2006). Voor de huidige studie gaan we uit van een conservatieve schatting van het effect: 10 bij dezelfde standaard deviatie. Uitgaande van gemiddeld 2 doofblinde ouderen per verzorgende, een intraklasse correlatie van hoogstens 0.3 en een uitval van 25%, zijn 112 doofblinde ouderen in totaal nodig.

De inclusie criteria zijn:

- Doofblindheid in de vorm van ernstige functionele beperktheid volgens de OESO-standaard (OECD, 2000)
- Leeftijd ouder dan 55 jaar
- Aanwezigheid van mantelzorger

De daadwerkelijke uitvoering van de interventie zal worden gevolgd door middel van een semi-gestructureerd dagboek en worden gebruikt voor procesevaluatie.
D) Gereed maken van ZIGdb voor grootschalige verspreiding en verspreiding via Internet.
E) Focusgroep onderzoek onder uitvoerders van interventie over factoren die interventie bevorderd dan wel belemmerd hebben. Hieruit zullen aanbevelingen voor implementatie worden afgeleid.

Operationalisatie

Achtergrondkenmerken

Cliënt
Leeftijd
Geslacht
Opleiding
Woonsituatie en verblijfsduur
Samenwonend
Burgerlijke status

Premorbide persoonlijkheid: NEO-FFI
Cognitie: CST
IADL
Comorbiditeit/voorgeschiedenis

Mantelzorger
Leeftijd
Geslacht
Opleiding
Samenwonend met client
Burgerlijke status

Professional
Opleiding
Functie
Leeftijd
Geslacht
Aantal jaren in dienst

Meetinstrumenten
Gekozen is voor primaire uitkomstmaten die het dichts liggen bij het doel van zelfmanagement. Waar mogelijk is gebruik gemaakt van meetinstrumenten die valide en betrouwbaar zijn en gevoelig voor verandering. De primaire effectmaat voor doofblinde ouderen is autonomie. De secundaire effectmaten zijn isolement, depressie en verpleeghuisopname. De primaire effectmaat voor de mantelzorger is gevoel van competentie. Verder zal arbeidssatisfactie van de van de professionele verzorgenden en begeleiders als secundaire effectmaat worden vastgesteld. Wij beschrijven de criteriummaat voor inclusie, de uitkomstmaten en de achtergrondkenmerken.

Criteriummaat voor inclusie

Doofblindheid
Functionele beperkingen op het gebied van horen en zien worden gemeten volgens een internationale standaard, de zogeheten OECD-indicator (Organisation for Economic Co-operation and Development). Bij beperkingen in het horen wordt gevraagd of iemand een gesprek kan volgen met één andere persoon of in een groep van drie of meer personen (zo nodig met hoorapparaat). Bij beperkingen in het zien gaat het erom of iemand kleine letters in de krant kan lezen of op een afstand van vier meter iemands gezicht kan herkennen (zo nodig met bril of contactlenzen).
De antwoordcategorieën op deze vragen zijn:
1. 'zonder moeite'
2. 'met enige moeite'
3. 'met grote moeite'
4. 'kan niet'.
Wie per deelonderwerp één of meer vragen heeft beantwoord met 'met grote moeite' of 'kan niet', wordt beschouwd als "ernstig functioneel beperkt" ofwel gehandicapt voor dit onderdeel.
Uitkomstmaten

Autonomie schaal
De autonomie schaal is oorspronkelijk opgesteld ter vaststelling van autonomie problemen en behoefte aan aandacht hiervoor in de palliatieve zorg. Het is een betrouwbaar en valide

instrument. Het instrument bevat negen vragen, o.a. continuering gangbare activiteiten, moeten overdragen van taken, afhankelijk voelen van anderen en ervaring van verlies van controle over eigen leven (Vernooij-Dassen e.a., 2005). Per item kan worden aangegeven of iets een probleem is (ja, enigszins, nee).

CES-D
Depressie wordt vastgesteld met de Center for Epidemiologic Depression Scale (CES-D). De Nederlandse vertaling van Beekman (1994) wordt hiervoor gebruikt. Het is een valide en betrouwbaar meetinstrument dat gevoelig is voor verandering. Het instrument omvat 20 vragen. De antwoordcategorien zijn zelden tot altijd. Score range is 0-60. Een hogere score betekent meer depressiever klachten.

Isolement
Isolement wordt vastgesteld met twee maten van sociaal functioneren: de omvang van het sociaal netwerk en contact frequentie. De omvang van het netwerk wordt vastgesteld met gestandaardiseerde vragen. De frequentie wordt bepaald met 6 items over sociale activiteiten, die worden gescoord op een 5-punts schaal, waarmee de frequentie van het contact wordt weergegeven (Wilson e.a., 2007).

Verpleeghuisopname
Datum opname.

Gevoel van competentie
Een 27-item schaal. De schaal is valide, betrouwbaar en gevoelig voor verandering (Moniz-Cook e.a, 2008:Vernooij-Dassen, 1996; 1999). Het instrument bevat drie domeinen : tevredenheid over zichzelf als verzorger, tevredenheid over patient als verzorgde, consequenties voor persoonlijk leven. De antwoorden worden gescoord op een 5-puntsschaal en varieren van helemaal mee eens tot helemaal mee oneens. De score range bedraagt 27-135. Een hogere score betekent een hoger competentie.

Arbeidssatisfactie
Arbeidssatisfactie wordt gemeten met de 'Job Stress and Satisfaction questionnaire' (Graham e.a., 2000), vertaald naar de Nederlandse situatie (Visser e.a, 2003). Het instrument bevat in totaal 47 items en 7 subschalen. Voorbeelden van subschalen zijn: zich gewaardeerd voelen, goede relaties met patiënt en familie hebben en zich goed toegerust voelen voor de taak. Het is een valide en betrouwbaar meetinstrument.

Achtergrondkenmerken

De NEO-Five Factor Inventory (NEO-FFI)
De NEO-FFI persoonlijkheidsvragenlijst meet de vijf belangrijkste domeinen van de persoonlijkheid bij volwassenen namelijk neuroticisme, extraversie, openheid, altruïsme en consciëntieusheid. Ieder domein bestaat uit 12 vragen, die gescoord worden op een vijfpuntsschaal. De totaalscores van elk domein worden omgezet in genormeerde scores naar geslacht of leeftijdscategorie. De afnameduur bij horende volwassenen vergt tien minuten. De NEO-FFI is betrouwbaar en valide voor oudere mannen en vrouwen.

Co-morbiditeit
Voorgeschiedenis/co-morbiditeit
Met behulp van een semi-gestructureerd interview met de doofblinde oudere en zijn mantelzorger worden relevante gegevens uit de voorgeschiedenis verzameld. Onder meer

wordt er gevraagd naar de oorzaken, tijdstip van ontstaan en verloop van de visuele en auditieve beperkingen. Co-morbiditeit wordt vastgesteld met de vertaalde Cumulative Sating Scale for Geriatrics (Miller, Paradis 1991: Graff e.a, 2006).

De Cognitieve Screeningstest (CST)
Met de CST wordt een globale indruk verkregen van de cognitieve domeinen oriëntatie en geheugen. Hij bevat 20 vragen. Het is een betrouwbaar en valide meetinstrument (Deelman, 1989)

IADL
IADL wordt gemeten met de Instrumental Activities of Daily Living – PSMS / IADL (Lawton & Brody, 1969).Dit is een veel gebruikt en valide meetinstrument (Moniz-cook et al, 2008: Lawton, Brody, 1969)

Setting, tijdsfasering en uitvoering

Setting:
Het onderzoek vindt plaats bij de oudere thuis en in het verzorgingshuis plaats in Nederland.

Tijdsfasering:
- Systematische literatuurstudies (maand1-4)
- Interviewguide (maand 5)
- Diepte interviews (maand 6-7)
- Maken van Zelfmanagement Interventie Gids doofblinden (ZIGdb) (maand 8-9)
- Pilotonderzoek toepasbaarheid en effecten van ZIGdb (maand 10-12)
- Randomised controlled trial naar effecten van ZIGdb op doofblinde ouderen en hun mantelzorgers: inclusie, voormeting, interventie, dagboeken, nameting (maand 13-36).
- Analyse (maand 37-41)
- Aanpassing voor grootschalige verspreiding (maand 42)
- Focusgroep grootschalige verspreiding (maand 43-46)
- Rapport (maand 47-48)

Totaal: 48 maanden

Tabel Variabelen en tijdsduur

**Variabelen en tijdsduur**	**Inclusie**	**Voor interventieperiode**			**Na interventieperiode**		
		DbO	Mz	Prof	DbO	Mz	Prof
Leeftijd, geslacht, opleiding, woonsituatie, burgerlijke status	X						
Voorgeschiedenis/comorbiditeit	X						
Visueel en auditief functioneren	X						
NEO-FFI (20 minuten)		X					
CST (15 minuten)		X					
IADL (10 minuten.)		X					
sPAQ (5 minuten)		X			X		
CES-D (10 minuten)		X			X		
Isolement (10 minuten)		X			X		
Verpleeghuisopname (notitie Mz)							X
Competentie mantelzorger(15 min)			X			X	
Arbeidssatisfactie(10 minuten.)				X			X

DbO = doofblinde oudere, Mz = mantelzorger, Prof = professionele verzorger, begeleider.

Uitvoering:
De vragenlijsten voor de doofblinden ouderen worden mondeling afgenomen door interviewers die bekend zijn met de zorg voor doofblinden. Het interview met de mantelzorger wordt eveneens mondeling afgenomen. De mantelzorger is ook de bron voor de meeste achtergrondkenmerken van de doofblinde oudere. Het tijdstip van eventuele opname wordt via de mantelzorger verzameld. De gegevens van de professionele verzorgenden en begeleiders worden schriftelijk verzameld.
De uitvoering van de interventies gebeurt door professionele verzorgenden en begeleiders. Logopedisten/communicatiedeskundigen gespecialiseerd in de doofblindenzorg scholen en coachen de professionele verzorgenden en begeleiders. De leerdoelen van de eendaagse scholing betreffen kennis en vaardigheden op het gebied van communicatie en omgang met doofblinde ouderen en vaardigheden op het gebied van zelfmanagement. Verbreding en verdieping van de competenties van de verzorgenden en begeleiders op deze gebieden wordt bereikt door maandelijkse coachingsessies van ieder anderhalf uur. De coaching vindt zowel individueel als in groepen van maximaal 4 deelnemers plaats.

Analyse
Het effect van de primaire uitkomstmaten zal worden berekend met de hiërarchische regressie analyse. Het effect op de secundaire uitkomstmaat opname in verpleeghuis zal worden bepaald met survival analyse (Kaplan-Meier). Cox-regressie analyse zal worden gebruikt om te corrigeren voor confounders. Proces analyse zal worden uitgevoerd om het daadwerkelijke gebruik van de interventie in kaart te brengen.

18. Verwachte resultaten
De verwachte effecten zijn verbetering van autonomie van de oudere met doofblindheid, verbeterde competentiegevoelens van de mantelzorgers en verbeterde arbeidssatisfactie van verzorgenden en begeleiders. Gekozen is voor primaire uitkomstmaten die het dichts liggen bij het doel van zelfmanagement. Daarnaast volgen wij ook de ontwikkeling van secundaire en meer indirecte uitkomstmaten en verwachten wij hierbij een vermindering van isolement en depressie bij oudere doofblinden en uitstel van verpleeghuisopname. Arbeidssatisfactie bij verzorgenden en begeleiders is gekozen als uitkomstmaat omdat zij bepalend is voor het stimuleren/afremmen van de regie van de oudere en van invloed zijn op uitstel van opname. De verwachting is dat het aanreiken van manieren om het zelfmanagement en met name de communicatie te bevorderen de arbeidssatisfactie zal vergroten.

19. Referenties

Beekman, J. Van Limbeek, D. Deeg. Een screeningsinstrument voor depressie bij ouderen in de algemene bevolking. Tijdschrift voor Gerontologie en Geriatrie, 1994, 25: 95-103.

Capella-McDonall, M.E.. The effect of single and dual sensory loss on symptoms of depression in the elderly. International Journal of Geriatric Psychiatry, 2005, 20: 855-861.

Deelman BG, Maring W, Otten V. De CST, een gestandaardiseerde screeningsmethode voor dementie. In: Schroots JJF e.a. Red. Gezond zijn is ouder worden. Assen/Maastricht: Van Gorcum, 1989, 163-70.

Graff MJL, Vernooij-Dassen, MJM, Thijssen M, Dekker J, Hoefnagels WHL, Olde Rikkert MGM. Effects of community occupational therapy on quality of life, mood and health status in dementia patients and their caregivers: a randomized controlled trial, Journals of Gerontology: Medical Science. J Gerontol A Biol Sci Med Sci. 2007; 62(9):1002-9.

Jee, J, Wang JJ, Rose KA, Lindley R, Landau P, Mitchell P. Vision and hearing impairment in aged care clients. Ophthalmic Epidemiol. 2005, 12(3): 199-205.

Kempen, G.I.J.M.. Preventie en ouder worden: de mogelijkheden voor zelfmanagement. Epidemiologisch bulletin, 2006, 2/3: 27-32.

Lawton, M.P. & Brody, E.M. (1969). Assessment of older people: self maintaining and instrumental activities of daily living. Gerontologist, 9, 179-189.

Lorig K.R., M.L. Hurwicz, D. Sobel, M. Hobbs, P.L. Ritter. A national dissemination of an evidence-based self-management program: a process evaluation study. Patient Education Counsultancy, 2005, 59(1):69-79.

Miller MD, Paradis CF. Rating chronic medical illness burden in geropsychiatric practice and research: application of the Cumulative Illness Rating Scale.
Psychiatry Res. 1992 Mar;41(3):237-48.

Moniz-Cook E, Vernooij-Dassen M, Woods R, Verhey F, Chattat R, de Vugt M, Mountain G, O'Connell M, Harrison J, Vasse E, Dröes RM, & Orrell M For the INTERDEM group. A European consensus on outcome measures for psychosocial intervention research in dementia care. Aging and Mental Health. Accepted for publication.

Organisation for Economic Co-operation and Development (OECD). A System of Health Accounts, 2000.

Vaal, J., J. Gusselkloo, M.M. De Klerk, D.H. Frijters, H.M. Evenhuis, A.P. Van Beek, R.M. Van Nispen, C. Smits, D.J. Deeg. Combined vision and hearing impairment: in an estimated 30 000- 35 000 people aged 55 years and over in The Netherlands. Nederlands Tijdschrift voor Geneeskunde, 2007, 30;151:1459-63.

Vernooij-Dassen, M. J., Felling, A. J., Brummelkamp, E., Dauzenberg, M. G., van den Bos, G. A. & Grol, R. (1999) Assessment of caregiver's competence in dealing with the burden of caregiving for a dementia patient: a Short Sense of Competence Questionnaire (SSCQ) suitable for clinical practice. *JAGS*, 47, 256-262.

Vernooij-Dassen, M., Lamers, C., Bor, J., Felling, A. & Grol, R. (2000). Prognostic factors of effectiveness of a support program for caregivers of dementia patients. *Int. J. Aging Hum.Dev., **51,*** 259-274.

Vernooij-Dassen M J FJ, Osse H P, Schade E, Grol R P T M. Autonomy problems and needs for care in cancer patients in the palliative phase of the disease. J Pain Symptom Manage 2005; 30:264-70.

Visser, M. R. M., Smets, E. M. A., Oort, F. J. & de Haes, H. C. J. M. (2003) Stress, satisfaction and burnout among Dutch medical specialists. *Canadian Medical Association Journal*, 168, 271-275.

Wilson, R., K. Krueger, S. Arnold, J. Schneider, J. Kelly, L. Barnes, Y. Tang, D. Bennett. Loneliness and Risk of Alzheimer Disease. Arch. Gen. Psychiatry, 2007, 64, 234-240.
