# Supplementary material for: Problems identified by dual sensory impaired older adults in long-term care when using a self-management program: A qualitative study
Source: PLoS One. 2017 Mar 21;12(3):e0173601. doi: 10.1371/journal.pone.0173601 (PMC5360251; doi:10.1371/journal.pone.0173601)
Supplement: S2 File — (DOCX) [file pone.0173601.s004.docx]

**Main points Ethical Approval**

The study design and protocol of the study ‘Self-Management for deafblind older adults and their informal caretakers: an effective approach?’ was reviewed by the Committee Research Involving Human Subject region Arnhem-Nijmegen.

Underneath the main points translated in English:

1. Translation of the approval-letter:

A copy of the original Dutch version can be found in the pdf-document Approval Med Eth that was added as Supporting Information, page 2.

2.

3. English summary of the application:
